# Radiation protection: safety measures and knowledge among interventional radiologists- a UK-based analysis of current practices and recommendations for improvement

**DOI:** 10.1186/s42155-025-00540-3

**Published:** 2025-04-22

**Authors:** Rayhan Y. Gasiea, Andy Rogers, Raghuram Lakshminarayan, Mo Hamady, Bella Huasen

**Affiliations:** 1 North West School of Radiology, Manchester, UK; 2 Nottingham University Hospitals NHS Trust, Nottingham, UK; 3 Hull University Teaching Hospital, Hull, UK; 4 Imperial College London, London, UK; 5 Lancashire Teaching Hospitals NHS Foundation Trust, Preston, UK

## Abstract

• Lack of Evidence-Based Practice: There is a significant gap in evidence-based practices related to radiation protection. Our understanding of global standards and practices, as well as their long-term impact on operator health, remains limited. Standardising current procedures and establishing a uniform database for data entry by all practitioners performing X-ray-based procedures would be beneficial.

• Lack of tailored measures to enhance protection for Female Operators: Female operators are at a greater risk of radiation exposure (Front Genet 10:260, 2019), underscoring the need for comprehensive guidance and standards to mitigate both short—and long-term health risks. Research and higher-level evidence are essential to evaluate the efficacy of current practices, along with better access to tools for accurate fetal dose calculation when needed.

• Balancing Protection and Ergonomics: It is crucial to achieve the right balance between adequate lead protection and ergonomic considerations for the spine and musculoskeletal system. The minimum level of lead protection scientifically required to ensure operator safety without compromising comfort and posture must be determined.

• Lack of accurate data regarding the log of health issues for personnel working with radiation gears. A national or international anonymised registry would be ideal for capturing and monitoring such data.

**Supplementary Information:**

The online version contains supplementary material available at 10.1186/s42155-025-00540-3.

## Background

The use of ionising radiation (X-rays) in medicine has increased over the past 20 years due to the introduction of new diagnostic and therapeutic practices. This has led to greater radiation exposure for healthcare workers, particularly in fields such as interventional radiology (IR), where complex procedures can involve high fluoroscopy times and dose rates, thereby delivering high radiation doses to both patients and medical staff [1–3].

Radiation exposure to staff and patients causes both deterministic and stochastic effects. The deterministic effects include cataracts, erythema of the skin, and sterility. Stochastic effects would involve risks related to DNA damage and thereby increase the risk of cancer and fetal malformations [4].

The International Commission on Radiological Protection (IRCP) has established dose limits for occupational exposure. The recommended limits are 20 millisieverts (mSv) per year, averaged over five years, while the annual occupational exposure limit should not exceed 50 mSv [5].

At the UK Health Security Agency (UKHSA), the International Commission on Radiological Protection (ICRP), and the United Nations Scientific Committee on the Effects of Atomic Radiation (UNSCEAR) Task Group, there is ongoing research into diseases of the nervous system. While the foundational science in this area is still relatively sparse, a growing body of evidence suggests that low dose exposures during prenatal or early childhood stages can have significant effects [6].

Current guidelines and safety measures in interventional radiology aim to limit radiation exposure as much as possible, emphasising the use of personal protective equipment (PPE), adhering to the principles of ALARA (As Low As Reasonably Achievable) and implementing shielding systems. Additionally, radiation protection is a significant component of the Royal College of Radiologists (RCR) curriculum, emphasised in the Fellowship of the Royal College of Radiologists (FRCR) Part A Physics exam [7]. System compliance, awareness, and knowledge among healthcare professionals are fundamental requirements for implementing effective radiation protection [5, 8, 9].

This real-time study was conducted to capture interventional radiologists' current knowledge and evaluate contemporary radiation protection measures in the United Kingdom. The study aimed to: 1) understand staff concerns regarding radiation protection 2) examine current practice and access to radiation protection tools and 3) appraise measures and adjustments designed to protect staff in general, particularly those who are pregnant.

## Methods

A questionnaire survey, based on an original one from 2017 by the same authors, was distributed to UK healthcare workers who worked with ionising radiation via the British Society of Interventional Radiology (BSIR) members’ registry. This group included trainees, fellows, consultants, nurses, and radiographers. The questionnaire gathered information on various areas, such as the provision and compliance of personal protective equipment (PPE), adverse symptoms related to PPE, dosimetry use, knowledge about radiation protection measures, regular occupational radiation protection training, and the receipt of individualised risk assessments and reasonable adjustments for pregnant individuals (See appendix).

Data collection adhered to general data protection regulations. The survey, provided in the appendix, was created using Survey Monkey® and distributed to the target population. The survey ran for 3 weeks during February 2024.

## Results

Percentages are rounded to two significant figures; therefore, totals may not sum to 100%.

## Demographics

**Fig. 1:**
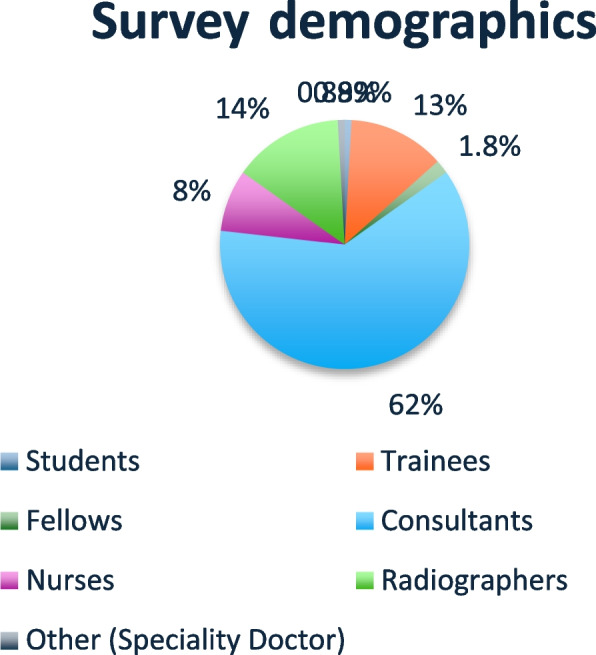
Survey demographics

Active members of BSIR (more than 1100) were contacted to complete the questionnaire. A total of 112 responses were received, categorised as follows: 0.89% (*n* = 1) were students, 13% (*n* = 14) were trainees, 1.8% (*n* = 2) were fellows, 62% (*n* = 69) were IR consultants, 8.0% (*n* = 9) were IR nurses, 14% (*n* = 16) were radiographers and 0.9% (*n* = 1) were other: speciality doctor (Fig. 1, Demographics).

### Duration of practice in IR

**Fig. 2:**
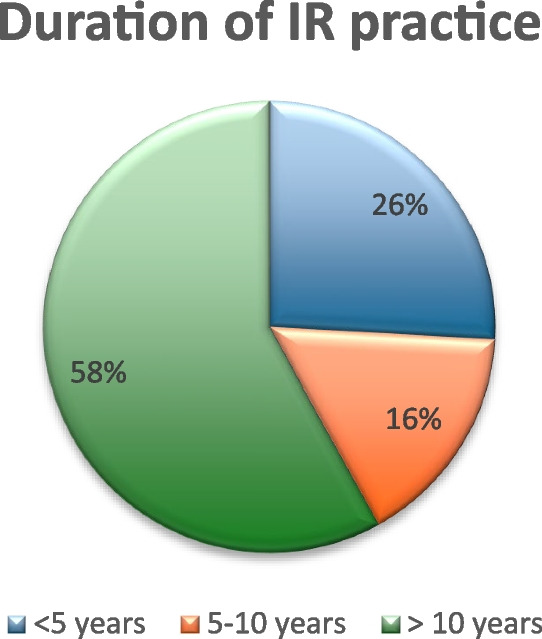
Duration of IR practice

When asked about the duration of practice in IR, 58% (*n* = 65) reported more than10 years duration in IR, 26% (*n* = 25) had 5–10 years, and 16% (*n* = 18) had less than 5 years of IR (Fig. 2, duration of practice in Interventional Radiology).

### Level of protection

**Fig. 3:**
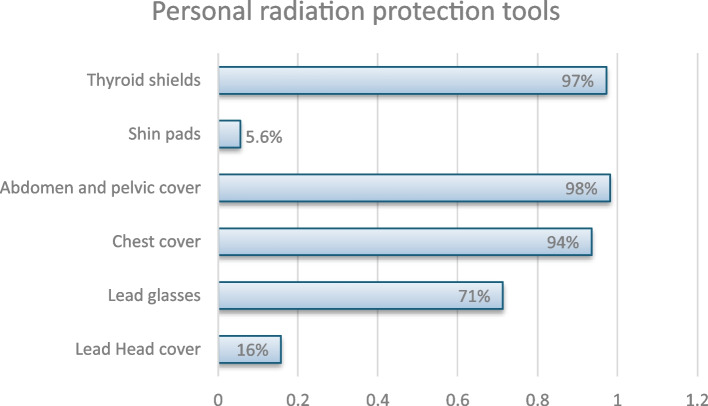
Personal radiation protection tools

Out of 112 respondents, 108 answered questions regarding the protection used in IR. In total, 97% (*n* = 105) used thyroid shields, 94% (*n* = 101) used chest cover, 98% (*n* = 106) used abdomen and pelvic cover, 16% (*n* = 17) used lead head cover, 71% (*n* = 77) used lead glasses and 5.6% (*n* = 6) used shin pads (Fig. 3, Level of protection).

Of 108 responding to the availability of lead screens/ceiling-mounted equipment in X-ray procedure rooms, 29 (26.9%) reported having two in all rooms, while another 29 (26.9%) had them in some rooms. In contrast, 38 (35.2%) reported having only one in all rooms, 9 (8.3%) had one in some rooms, and 3 (2.8%) had none. Regarding lead skirts, 84 (77.8%) reported having them in all rooms, 23 (21.3%) in some rooms, and 1 (0.9%) in none.

### Monitoring/dosimeter tools

**Fig. 4:**
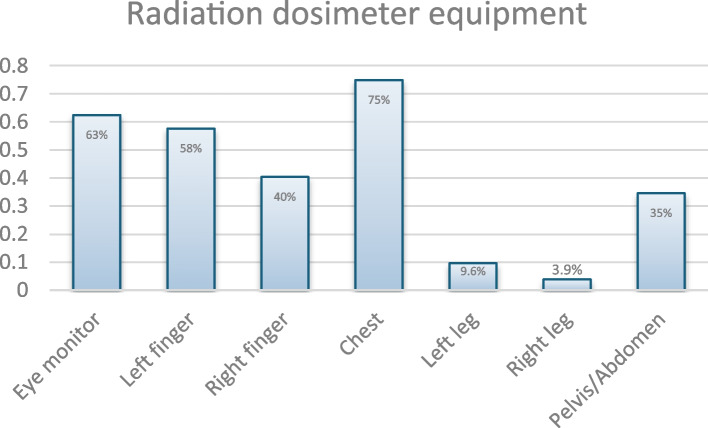
Radiation dosimeter equipment

A total of 104 respondents answered the question regarding the dosimeter tools used. Among them, 63% (*n* = 65) reported utilising eye monitors, 58% (*n* = 60) employed a left finger monitor, 40% (*n* = 42) used a right finger monitor, 75% (*n* = 78) relied on a chest monitor, 9.6% (*n* = 10) opted for a left leg monitor, 3.9% (*n* = 4) selected a right leg monitor, and 35% (*n* = 36) made use of a pelvis/abdomen monitor (Fig. 4, Monitoring/dosimeter tools).

Out of 104 respondents, 78 individuals (75%) indicated changing their dosimeters monthly, while 26 individuals (25%) reported changing them quarterly. Among 105 respondents, 66 individuals (62.9%) stated they had never delayed or missed a dosimeter change in the past six months. Additionally, 25 individuals (23.8%) admitted to forgetting to change their dosimeter for less than a week. In comparison, six individuals (5.7%) forgot for less than a month, and eight (7.6%) reported forgetting for over a month.

### Reported health issues since working in IR

**Fig. 5:**
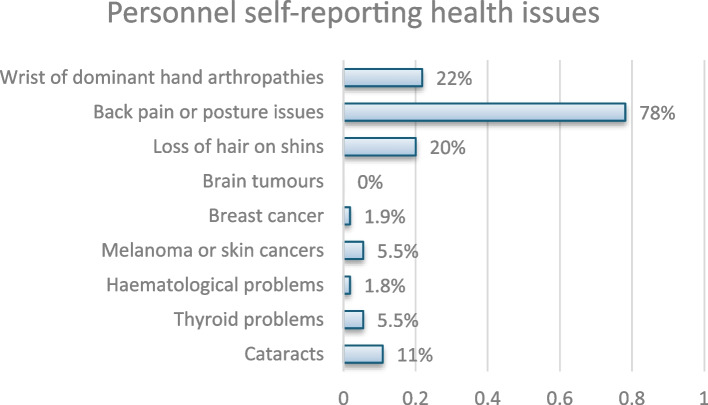
Personnel self-reporting health issues

Among the 55 respondents to the question, 22% (*n* = 12) reported arthropathies in the wrist of their dominant hand, 78% (*n* = 43) reported back pain or posture issues, 20% (*n* = 11) reported hair loss on their shins, none reported brain tumours, 1.8% (*n* = 1) reported breast cancer, 5.5% (*n* = 3) reported melanoma or skin cancers, 1.8% (*n* = 1) reported haematological problems, 5.5% (*n* = 3) reported thyroid issues, and 11% (*n* = 6) reported cataracts (Fig. 5, Reported health issues since working in IR). Out of 105 respondents, 39 (37.1%) reported having an annual eye check, while 66 (62.9%) did not report having one.

### Adequacy of radiation protection and radiation protection training

**Fig. 6:**
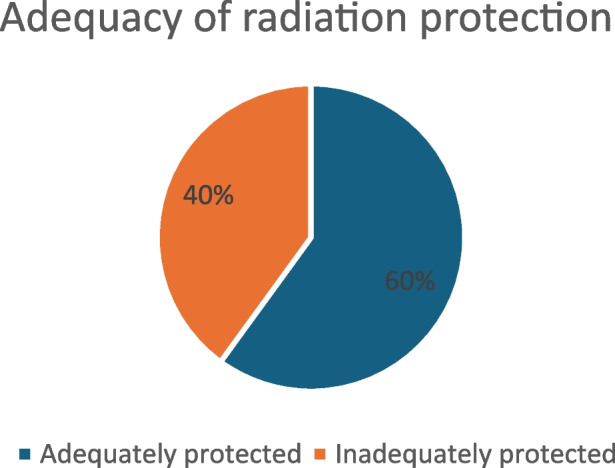
Adequacy of radiation protection

**Fig. 7:**
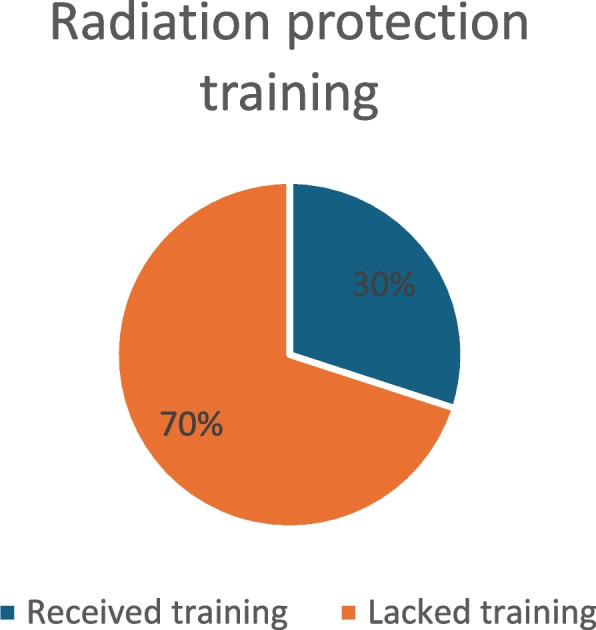
Radiation protection training

Approximately 40% felt inadequately protected (Fig. 6, Adequacy of Radiation Protection) and 70% reported a lack of training in radiation protection (Fig. 7, Radiation Protection Training).

### Concerns regarding radiation-related risks

**Fig. 8:**
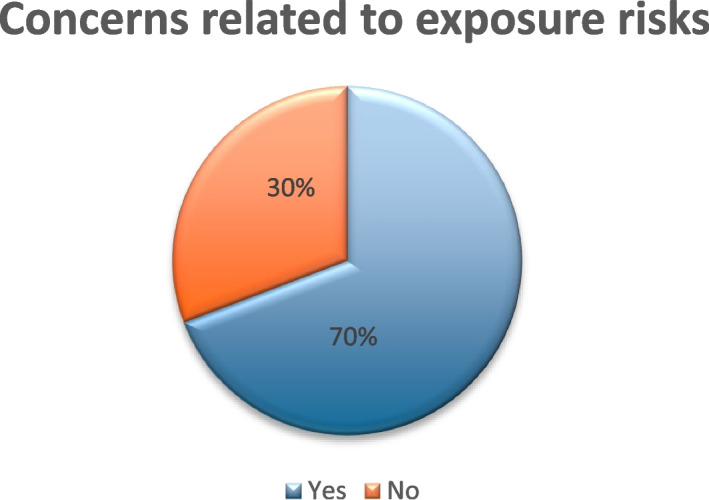
Concerns related to exposure risks

A total of 104 out of 112 individuals responded to this question. Among the respondents, 69% (*n* = 72) expressed concerns about radiation-related risks (Fig. 8, concerns regarding radiation-related risks).

### Reasonable adjustments during pregnancy

**Fig. 9:**
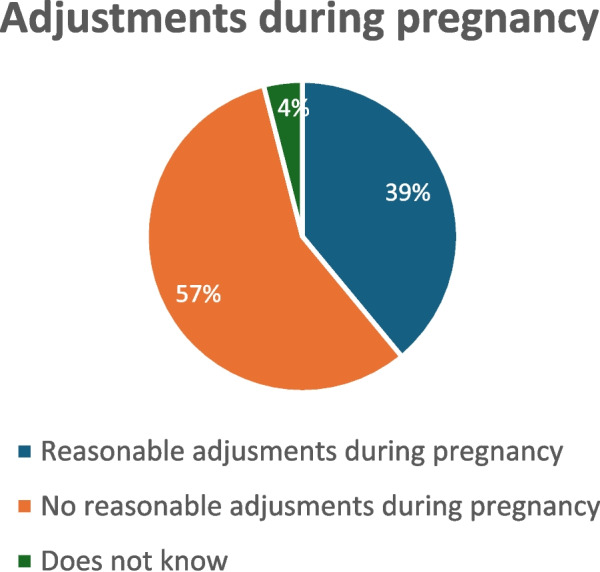
Adjustments during pregnancy

In total, 23 individuals commented on this topic. From this group, 39% (*n* = 9) indicated they received reasonable adjustments during pregnancy, while 57% (*n* = 13) stated they did not receive any adjustments during pregnancy (Fig. 9, reasonable adjustments during pregnancy). Additionally, 4% (*n* = 1) responded that they did not know.

**Fig. 10:**
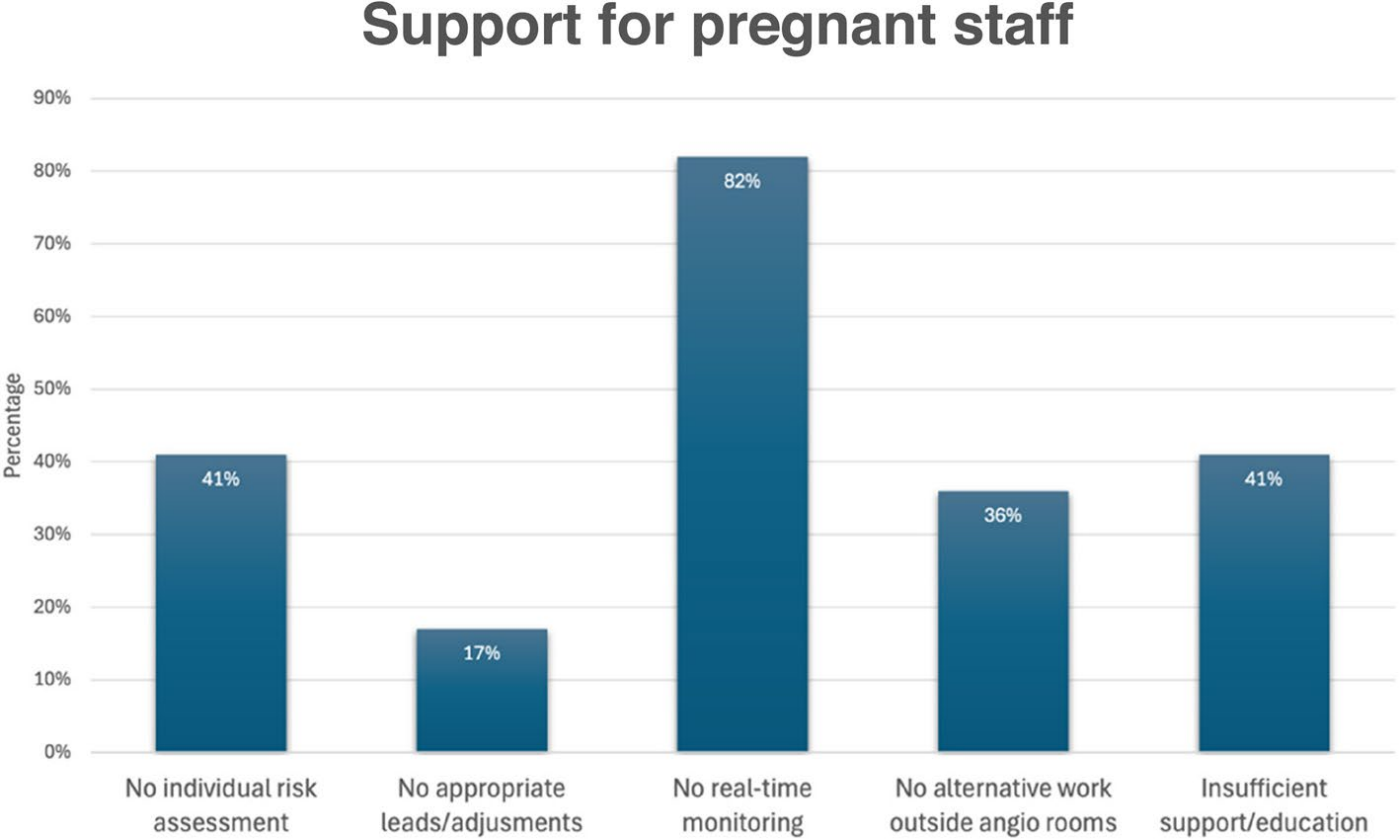
Support for pregnant staff

The survey revealed significant gaps in support (Fig. 10, Support and accommodation during pregnancy) and accommodations for pregnant staff in the interventional radiology setting. Specifically, 41% of participants reported not being offered individualised risk assessments, 17% lacked appropriate leads or adjustments, and 82% did not receive real-time monitoring during complex procedures. Additionally, 36% were not provided with alternative work outside angio rooms to reduce radiation exposure, and 41% indicated insufficient support or education.

### Radiation exposure and safety awareness

**Fig. 11:**
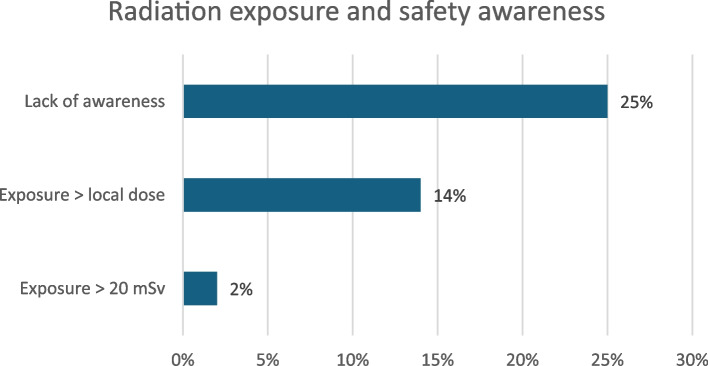
Radiation exposure and safety awareness

Among respondents, 2% reported radiation exposure exceeding the 20 mSv dose, while 14% exceeded the local departmental dose limits. Additionally, 25% indicated a lack of awareness regarding their exposure levels (see Fig. 11 for details on radiation exposure and safety awareness).

### Percentage of designated lead gear

**Fig. 12:**
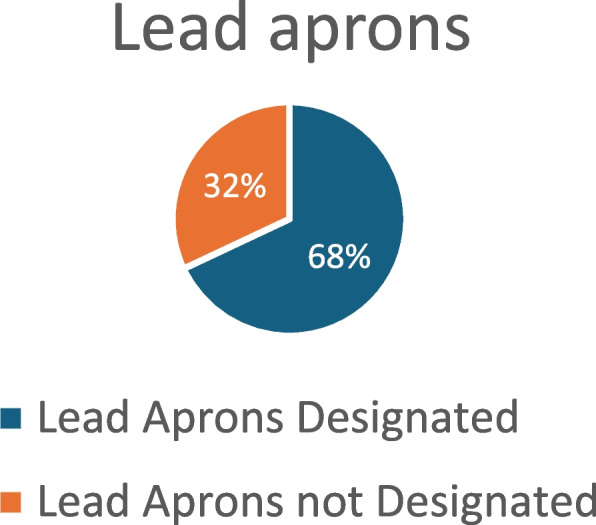
Lead aprons

**Fig. 13:**
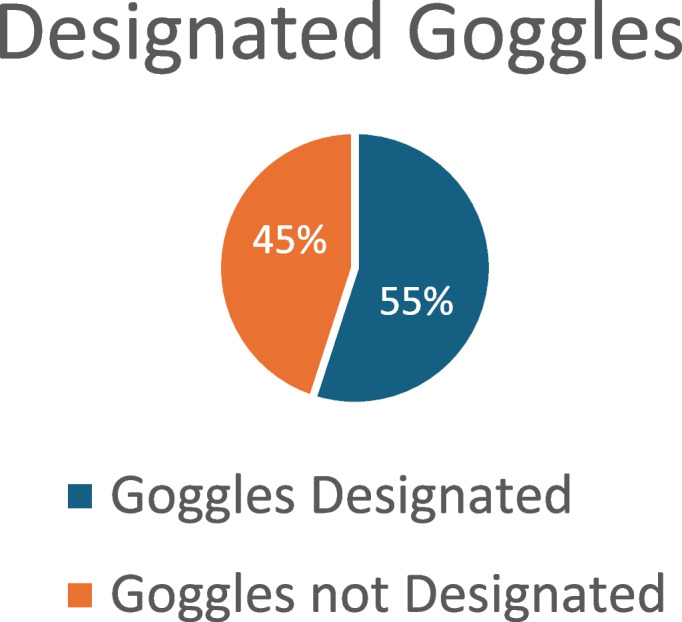
Designated Goggles

The survey found that 68% of respondents had lead aprons specifically designated for their use, while 55% had individually designated lead goggles (Fig. 12, lead apron and Fig 13, Goggles).

## Discussion

The current survey, a questionnaire involving diverse healthcare providers practising in interventional radiology (IR) settings within the UK, sheds light on radiation safety practices and the challenges faced by practitioners across various levels and domains. The initiative began in 2017 with a SurveyMonkey questionnaire titled *Perceptions and Misconceptions of IR*, which surveyed female medical students and trainees about their views on pursuing a career in IR. Concerns regarding radiation protection were subsequently raised during the Asia Pacific Society of Cardiovascular and Interventional Radiology (APSCVIR) Annual Scientific Meeting in Auckland in 2018. This led to a publication [10] that examined career entry barriers and addressed misconceptions about radiation exposure during pregnancy, which were found to discourage women from entering the field. In response, the authors launched awareness campaigns in 2023, including a YouTube video [11] addressing the dangers of ionising radiation, and expanded their efforts with the current survey via BSIR to include participants of all genders and experience levels.

The survey highlighted that nearly 70% of multidisciplinary respondents have concerns about radiation-related risks. Additionally, close to 40% of respondents felt inadequately protected, while approximately 70% lacked up-to-date radiation protection training.

While a small percentage (2%) of participants reported exposure exceeding the 20 mSv dose limit, the potential gaps in dose monitoring and adherence to safety protocols remain a significant concern. Approximately a quarter of respondents indicated they were unaware of their radiation exposure levels. These findings underscore the necessity of enhancing radiation safety records and raising awareness among healthcare providers in IR settings.

Participants reported widespread use of radiation protection gear, including thyroid shields (97.2%), chest covers (93.5%), and abdomen and pelvic covers (98.2%). However, fewer participants used lead glasses (71.3%), and head covers (15.7%), indicating a gap in eye and head protection. This is particularly concerning because sensitive areas like the eyes should be protected [12, 13]. In fact, in 2017, the eye lens dose limit was reduced from 150 to 20 mSv per year, highlighting the risks associated with radiation exposure, such as the formation of cataracts [14].

Overall, approximately half of the respondents expressed concerns about their personal radiation protection equipment (53.4%). Personalised lead aprons, lead goggles, and prescription lead glasses were unavailable to 34%, 46%, and 40% of respondents. It is particularly concerning that trainees reported a lack of access to funded personal PPE despite their heavy IR commitment of 5–7 sessions per week.

Whilst we lack registries or systems to log health complications of staff during their X-RAY led work life, there is lack of reporting of incidents and sometimes unconscious lack of association with scatter exposure. Amongst 100 participants 11% reported cataracts, 20% hair loss on shins, 80% MSK issues, 22% dominant wrist/hand arthropathies, 6% melanoma/skin cancers, 6% thyroid problems and 2% haematological problems. These challenges are consistent with the findings of Ploussi and Efstathopoulos [2], highlighting the critical need for improved reporting mechanisms and greater awareness among healthcare professionals regarding potential health hazards.

We realised early on during interviews and surveys that many clinicians are apprehensive about documenting or mentioning health problems. They often do not associate the health issues encountered with working in radiation and, as a result, fail to report them.

The trainee survey revealed notable health issues, with more than 90% of respondents reporting back pain, nearly 40% experiencing neck pain, and approximately 15% encountering hip and knee pain linked to their personal protective equipment (PPE).

Heavy leaded aprons, a common practice in our work environment, have been associated with orthopaedic injuries and musculoskeletal pain [15]. To address these issues, advancements in new shielding systems have been designed to mitigate the orthopaedic risks associated with traditional PPE. While these systems show promise, with studies reporting a 60% reduction in radiation with the Rampart system [16] and significant exposure reduction with the Protego system, including no exposure in 68% of cases [17], they also have limitations. For instance, studies have revealed limited shielding against left-side radiation [18] and noted a reduced radiation reduction for positions further from the radiation source [16]. Therefore, more research is required to fully understand these shielding systems' advantages and limitations.

Worryingly, our findings reveal significant gaps in the support and accommodations provided to pregnant staff members. Approximately, 41% of pregnant employees were not offered individualised risk assessments, and 16.67% were not provided with appropriate leads or adjustments. Moreover, 82% were not offered real-time monitoring, particularly concerning complex procedures. Additionally, 36% of pregnant staff were not offered alternative work outside the angio rooms, which could potentially minimise radiation exposure risks. Furthermore, 41% of pregnant employees reported insufficient support or education.

This situation is particularly concerning given the importance of monitoring radiation exposure for pregnant employees to ensure compliance with UK regulations. As highlighted by Yoon and Slesinger (2023) [19], it is crucial to monitor occupational radiation exposure for pregnant employees to keep the total amount of radiation exposure below the regulatory limit. In the UK, the Ionising Radiations Regulations 2017 (IRR17) emphasise the ALARA (As Low As Reasonably Achievable) principle to minimize radiation exposure, especially for pregnant workers. While IRR17 does not set specific dose limits for the embryo/fetus, guidance aligns with recommendations from the Health Protection Agency and the International Commission on Radiological Protection (ICRP). The dose limit for the embryo/fetus is typically considered to be 1 mSv over the course of the pregnancy, with no single month exceeding 0.5 mSv to ensure that radiation exposure remains as low as reasonably achievable. Addressing these gaps in support and monitoring is essential to safeguard the health and well-being of pregnant staff members and their unborn children.

In Europe, there is a spectrum of restrictions regarding pregnant workers in the IR suite, ranging from a complete legal prohibition (in Italy and Portugal) to dose limits for the fetus (e.g., Spain) and the right to be reassigned (Sweden). The UK position mirrors the experiences in Spain and Sweden. The authors do not intend to suggest prohibition but aim to minimise exposure and risk through education and training to acceptable levels for pregnant IR staff. We suggest that CIRSE, as a leading organisation in intervention practice, should take the lead in creating comprehensive guidelines for IR practitioners across Europe and beyond.

Addressing the identified gaps in radiation safety practices requires a multifaceted approach encompassing education, training, and support initiatives. Enhanced awareness campaigns and targeted training programs can help improve healthcare providers’ knowledge and adherence to radiation safety protocols. Moreover, institutions must prioritise the safety and well-being of pregnant staff by implementing tailored risk assessment protocols and providing necessary support and accommodations. By addressing these challenges and implementing targeted interventions, healthcare institutions can enhance radiation safety practices in IR settings, ultimately ensuring the continued safety and well-being of staff members [20].

Currently, in the UK, there is no specific additional radiation protection syllabus for radiologist trainees pursuing IR career before their FRCR award. Given the responses to this survey, there is a clear need to develop guidance for all trainee IR staff, potentially drawing on the experience of the United States [21].

The study's findings are subject to limitations inherent in survey-based research, including potential response bias and inaccuracies in self-reporting. Furthermore, the sample size and demographics may not fully represent the broader population of healthcare providers in interventional radiology (IR) practice. Although the survey focused on UK IR settings, there is no reason to believe that the situation is significantly different in other European countries. The Cardiovascular and Interventional Radiological Society of Europe (CIRSE) is well-positioned to lead further research endeavours and educational initiatives that explore variations in practice and assess the effectiveness of interventions aimed at improving radiation safety practices in IR settings.

This study has some limitations. The number of respondents was relatively small compared to the number of IR practitioners in the UK. However, the collected responses and the range of roles provide a reasonable cross-sectional insight into radiation protection issues. The nature of the survey does not allow for exploring individual excessive exposure to ionising radiation or the actual implicated risks in more depth. Additionally, we could not identify areas of poor practice or examples of good practice for broader benefits.

## Conclusion

Healthcare professionals who use x-ray guidance to perform minimally invasive procedures can have healthy and fulfilling careers with minimal harm related to occupational radiation and the weight of protective garments. This can be achieved by raising awareness of scatter radiation, tailored to specific patient groups and procedures, and by addressing the appropriate radiation protection gear for individuals, along with considerations for musculoskeletal health and balance. More research, studies, and collaborative efforts are needed, particularly for the current generation in practice. A universal anonymised database where individuals working with ionising radiation can log health issues throughout their careers could help illuminate risks and generate more accurate data than historical estimates.

## Supplementary Information

Supplementary Material 1: Appendix. Survey Questionnaire.

## Data Availability

The survey results data are available from the corresponding author upon reasonable request.
