# Supplementary material for: Radiation protection: safety measures and knowledge among interventional radiologists- a UK-based analysis of current practices and recommendations for improvement
Source: CVIR Endovasc. 2025 Apr 22;8:32. doi: 10.1186/s42155-025-00540-3 (PMC12014967; doi:10.1186/s42155-025-00540-3)
Supplement: Supplementary file 1 — Supplementary Material 1: Appendix. Survey Questionnaire. [file 42155_2025_540_MOESM1_ESM.docx]

**Appendix**

**Survey Questionnaire**

Q1. What is your level of practice?

- Student
- Trainee
- Fellow
- Consultant
- Nurse
- Radiographer
- Other (please specify)

Q2. Where is most of your practice located?

- Angio room
- Multipurpose fluoroscopy room / C Arm
- Hybrid
- Combined Angio and hybrid rooms
- CT room
- All of the above

Q3. How long have you been in IR practice?

- < 5 years
- 5-10 years
- 10 years

Q4. What radiation protection gear do you use currently? Tick all that are

relevant

- Lead head cover
- Lead glasses
- Thyroid shield
- Chest cover
- Abdomen and pelvic cover
- Shin pads

Q5. Is your lead apron... (tick all that are relevant)?

- Wrap around
- Front and back only
- One piece
- Two pieces

Q6. Are the lead aprons specific to you / your body?

- YES
- NO

Q7. Are the goggles specific to you?

- Yes
- Yes, prescription
- No

Q8. Do you have 2 lead screens / ceiling mounted in all rooms used to

operate with XRAY procedures?

- Yes, 2 in all rooms
- Yes, 2, but only in some of the rooms
- No, 1 in all rooms
- No, 1, but only in some of the rooms
- None

Q9. Do you have a lead skirt attached to the table in all rooms used to

operate with XRAY procedures?

- Yes, all of the rooms
- Some of the rooms
- No, none of the rooms

Q10. Which monitoring / dosimeter tools do you currently use? Tick all that

are relevant

- Eye monitor
- Left finger
- Right finger
- Chest
- Left leg
- Right leg
- Pelvis / abdomen

Q11. How frequently are you asked to change the dosimeters?

- Monthly
- Quarterly
- No fixed time

Q12. In the last 6 months, have you delayed or not changed your

dosimeter?

- Never
- Forgot (< 1 week)
- Forgot (< 1 month)
- Forgot (> 1 month)

Q13. Are you sent a copy of your doses for your record?

- YES
- NO

Q14. Do you perform long and/or potentially high radiation dose

procedures?

- YES
- NO
- SOMETIMES

Q15. Have you ever exceeded the yearly dose / local limits?

- Yes, exceeded year dose 20msv
- Yes, exceeded the local investigation level
- No
- Do not know

Q16. If yes, what measures were taken?

Open answer

Q17. Do you have a yearly eye check?

- YES
- NO

Q18

Have you ever been diagnosed with any health problems that may be

related to radiation work?

- YES
- NO

Q19. If yes, please could you share brief information?

Open Answer

Q20. Have you suffered from any of the following since working in IR? Tick

all that are relevant.

- Cataracts
- Thyroid problems
- Haematological problems
- Melanoma or skin cancers
- Breast cancer
- Brain tumours
- Loss of hair on shins
- Back pain or posture issues
- Wrist of dominant hand arthropathies

Q21. Are you concerned about radiation related risks?

- YES
- NO

Q22. Do you feel adequately protected from scatter and radiation?

- YES
- NO

Q23. Do you know the name of the RPS and RPA for your department?

- YES
- NO

Q24. Do you think that you have an up-to-date knowledge about radiation

protection measures?

- YES
- NO
- SOMEWHAT

Q25. Are you offered regular occupational radiation protection training?

- YES
- NO

Q26. If yes, do you always attend?

- YES
- NO

Q27. Are you offered regular patient radiation protection training (IRMER

updates)?

- YES
- NO

Q28. If yes, do you always attend?

- YES
- NO

Q29. Did you receive an individualised risk assessment at your work when

pregnant?

- YES
- NO
- I DON'T KNOW
- N/A

Q30. Were you offered a bespoke lead or reasonable adjustments whilst pregnant?

- YES
- NO
- I DON'T KNOW
- N/A
